# Exploring Potential Biomarkers, Ferroptosis Mechanisms, and Therapeutic Targets Associated with Cutaneous Squamous Cell Carcinoma via Integrated Transcriptomic Analysis

**DOI:** 10.1155/2022/3524022

**Published:** 2022-09-19

**Authors:** Wenxing Su, Biao Huang, Qingyi Zhang, Wei Han, Lu An, Yi Guan, Jiang Ji, Daojiang Yu

**Affiliations:** ^1^Department of Plastic and Burn Surgery, The Second Affiliated Hospital of Chengdu Medical College (China National Nuclear Corporation 416 Hospital), Chengdu, Sichuan, China; ^2^Laboratory of Stem Cell and Tissue Engineering, Orthopedic Research Institute, Department of Orthopedics, West China Hospital of Sichuan University, Chengdu, Sichuan, China; ^3^Department of Burn and Plastic Surgery, The First Affiliated Hospital of Soochow University, Suzhou, Jiangsu, China; ^4^Department of Plastic Surgery, The Second Affiliated Hospital of Soochow University, Suzhou, Jiangsu, China; ^5^School of Foreign Languages, Soochow University, Suzhou, Jiangsu, China; ^6^Department of Dermatology, The Second Affiliated Hospital of Soochow University, Suzhou, Jiangsu, China

## Abstract

**Background:**

Cutaneous squamous cell carcinoma (cSCC) is the leading cause of death in patients with nonmelanoma skin cancers (NMSC). However, the unclear pathogenesis of cSCC limits the application of molecular targeted therapy.

**Methods:**

Three microarray datasets (GSE2503, GSE45164, and GSE66359) were downloaded from the Gene Expression Omnibus (GEO). After identifying the differentially expressed genes (DEGs) in tumor and nontumor tissues, five kinds of analyses, namely, functional annotation, protein-protein interaction (PPI) network, hub gene selection, TF-miRNA-mRNA regulatory network analysis, and ferroptosis mechanism, were performed.

**Results:**

A total of 146 DEGs were identified with significant differences, including 113 upregulated genes and 33 downregulated genes. The enriched functions and pathways of the DEGs included microtubule-based movement, ATP binding, cell cycle, *P53* signaling pathway, oocyte meiosis, and *PLK1* signaling events. Nine hub genes were identified (*CDK1*, *AURKA*, *RRM2*, *CENPE*, *CCNB1*, *KIAA0101*, *ZWINT*, *TOP2A*, and *ASPM*). Finally, *RRM2*, *AURKA,* and *SAT1* were identified as significant ferroptosis-related genes in cSCC. The differential expression of these genes has been verified in two other independent datasets.

**Conclusions:**

By integrated bioinformatic analysis, the hub genes identified in this study elucidated the molecular mechanism of the pathogenesis and progression of cSCC and are expected to become future biomarkers or therapeutic targets.

## 1. Background

Cutaneous squamous cell carcinoma (cSCC) is a type of malignant tumor that originates from the epidermis or appendage keratinocytes, with an incidence only second to basal cell carcinoma (BCC), accounting for approximately 20% of all nonmelanoma skin cancers (NMSC) [1, 2]. Recent studies have shown that somatic mutations in cSCC are much more frequent than in other squamous cell carcinomas [3], indicating a complex genetic background of cSCC, and suggesting that its pathogenesis may involve diverse genes and pathways. *P53*, *CDKN2A*, *NOTCH1,* and *NOTCH2* are the most commonly mutated genes [4, 5]. It has been confirmed that 54–95% of cSCC contains UV radiation-induced *P53* mutations [6]. Immunohistochemistry (IHC) also revealed that the expression of *P53* was closely related to the histological grade and TNM stage of cSCC [7]. Meanwhile, tumors with high *P53* protein expression are more aggressive than tumors with low *P53* protein expression [7]. Furthermore, cSCC often has heterozygous deletions or point mutations in the *CDKN2A* gene locus, and the deletion of *p16INK4a* is thought to be related to the progression of actinic keratosis (AK) to cSCC [8]. *NOTCH* is a direct target of *P53*, and more than 75% of cSCCs have *NOTCH1* and *NOTCH2* mutations [9]. However, the driving genes are not yet clear. It is thus significant to understand the exact molecular mechanisms underlying cSCC development, progression, and recurrence.

In recent years, gene expression analysis has provided an effective global method for elucidating the pathogenesis of many cancers, including skin cancer. However, the results of a single-chip data analysis are often unconvincing. In this study, therefore, three independent microarray datasets were downloaded from the GEO to obtain their common DEGs between cSCC and normal *epidermis*. Subsequently, we enriched the functions of these differential genes, constructed their PPI network, and selected the most important hub genes in the network. In summary, the DEGs and hub genes identified in this study may elucidate the molecular mechanism of the pathogenesis and progression of cSCC and are expected to become future biomarkers or therapeutic targets.

## 2. Methods

### 2.1. Raw Data Collection

GEO (https://www.ncbi.nlm.nih.gov/geo) [10] is a public database with free access to microarray data. We searched for related gene expression datasets using cutaneous squamous cell carcinoma as a keyword. The inclusion criteria were set as follows: the number of genes detected by the gene chip should be greater than 20,000 to obtain common DEGs, and the tested specimens included should be from humans. In addition, the number of genes detected in the validation set should be larger than that in the training set to prevent information about the hub genes from being unavailable. Three gene expression datasets (GSE2503 [11], GSE45164 [12] and GSE66359 [13]) were downloaded from it as the training set. In addition, the gene expression datasets (GSE53462 [14] and GSE7553 [15]) were downloaded as the validation set.  Table 1 shows the details of the five datasets.

### 2.2. Identification of DEGs

The DEGs between cSCC and noncancerous samples were screened using GEO2R (https://www.ncbi.nlm.nih.gov/geo/geo2r), which is an online tool for differential analysis of the original dataset based on the LIMMA software package [16]. A logFC (fold change) ≥ 1 and *p* value <0.05 were considered statistically significant.

### 2.3. Enrichment Analyses of DEGs

Gene Ontology (GO) analysis including biological processes (BP), cellular components (CC), and molecular functions (MF), was first performed to identify the unique biological characteristics of the DEGs. Then, the Kyoto Encyclopedia of Genes and Genomes (KEGG) pathway analysis was used to explore the main pathways involved in the occurrence and development of cSCC. Both enrichment results of the GO function and KEGG pathway were obtained from DAVID 6.8 (https://david.ncifcrf.gov/), which is an online functional annotation tool [17]. A *p* value < 0.05 was considered statistically significant.

### 2.4. PPI Network Construction and Module Analysis

The STRING database (https://string-db.org/; version 11.0) was used to explore the interactions of the DEGs [18]. A comprehensive score > 0.4 was selected to construct a PPI network and was visualized with Cytoscape software (version 3.7.2). Then, the most important functional modules in the PPI network were obtained through the plug-in MCODE in Cytoscape using the parameters of MCODE scores >10, degree cutoff = 2, node score cutoff = 0.2, k-score = 2, and max depth = 100. Subsequently, the important biological pathways, that this, module participates in were obtained through FunRich, which is a free software package that can perform functional enrichment analysis of genes or proteins [19].

### 2.5. Hub Gene Selection and Analysis

The nine genes with the highest degree of connectivity in the above modules were selected as hub genes. Their pathway analysis was performed and visualized by ClueGO (version 2.5.4) and CluePedia (version 1.5.4). A *p* value < 0.05 was considered statistically significant. An interaction network between the hub genes and their coexpressed genes was created using GeneMANIA (https://www.genemania.org/) [20], which is a convenient web portal for analyzing gene lists and predicting gene function. The DGIdb database (https://www.dgidb.org/) can be used to generate hypotheses about how genes can be targeted for therapy or prioritized for drug development [21]. In this study, the gene-drug interaction relationship was obtained through DGIdb 3.0 and visualized by Cytoscape. The parameters were: preset filters; FDA approved; antineoplastic; all default.

### 2.6. Validation of Hub Genes in Other Databases and the Human Protein Atlas

To confirm the reliability of our results, hub gene expression was verified in the GSE53462 and GSE7553 datasets by Student's *t*-test. A *p* value <0.05 was considered statistically significant. To further validate our findings, we searched the Human Protein Atlas (https://www.proteinatlas.org/) website for the immunohistochemical staining results of nine hub genes in normal skin and tumor tissue.

### 2.7. TF-miRNA-mRNA Regulatory Network Analysis

To further understand the regulatory mechanism of the hub genes, TF-target interactions were obtained through the Transcriptional Regulatory Relationships Unraveled by Sentence-based Text mining (TRRUST) [22], which is a database for the prediction of transcriptional regulatory networks, which contains the target genes corresponding to TFs and the regulatory relationships between TFs. In addition, miRNA-target interactions were obtained by Mirwalk [23], which is a publicly available database that focuses on miRNA -target interactions. To improve the accuracy, the predicted miRNAs that had been verified by experiments and other databases were screened. Finally, miRNA-target interactions and TF-target interactions were integrated to construct the TF-miRNA-mRNA regulatory network by Cytoscape.

### 2.8. Identification and Validation of Ferroptosis-Related DEGs in cSCC

A total of 259 ferroptosis-related genes were obtained from the Ferroptosis Database (https://www.zhounan.org/ferrdb) [24], and we intersected these genes with the DEGs of cSCC to screen ferroptosis-related genes in cSCC. To ensure the rigor and accuracy of this study, we verified the expression of these genes in GSE53462 and GSE7553. The comparison between the cSCC and control sets of data was performed by the Student's *t*-test, and *p* value < 0.05 was considered to be statistically significant. Based on the validation results, we removed the disqualified genes and finally obtained accurate ferroptosis-related genes involved in cSCC.

## 3. Results

### 3.1. Identification of DEGs in cSCC

**Figure 1:**
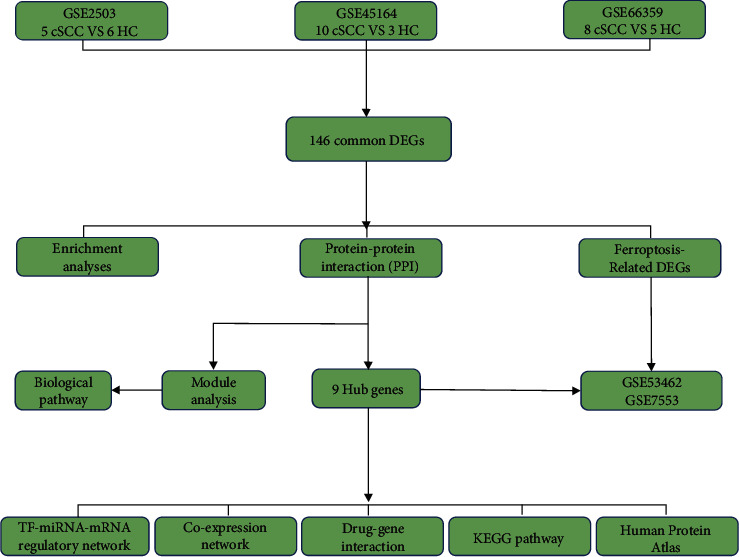
The design flow chart of this study.

**Figure 2:**
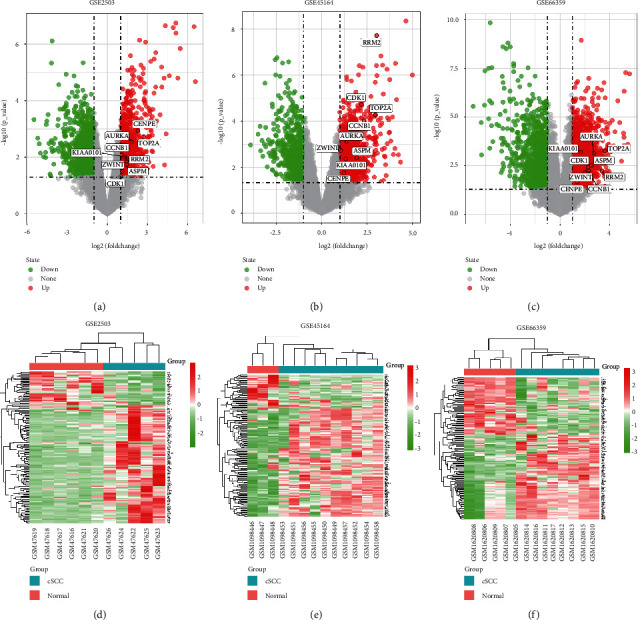
Differentially expressed analysis of GSE2503, GSE45164, and GSE66359 datasets. (a–c) was the differentially expressed volcano figure. (d–f) were heat maps of DEGs. Among them, red indicates upregulated genes and green indicates downregulated genes. Gray indicates no differential expression.

**Figure 3:**
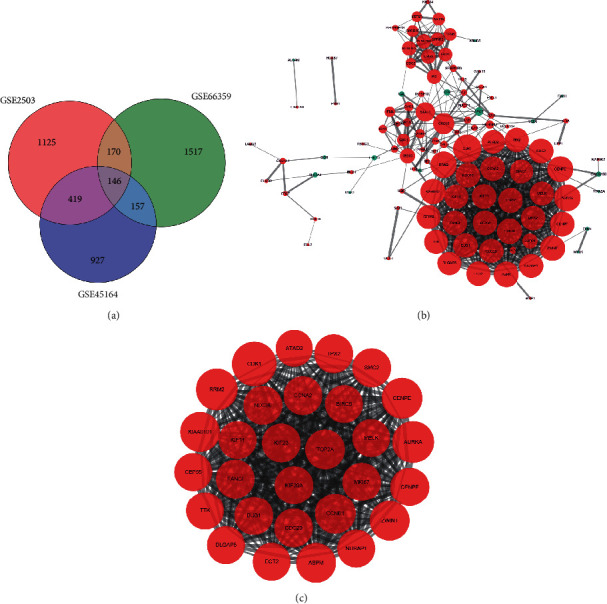
Venn diagram, PPI network, and the most significant module of DEGs. (a) DEGs were selected with a fold change > 1 and *p* value < 0.05 among the mRNA expression profiling sets GSE2503, GSE45164 and GSE66359. The 3 datasets showed an overlap of 146 genes. (b) The PPI network of DEGs was constructed using Cytoscape. (c) The most significant module was obtained from the PPI network with 29 nodes and 404 edges. Upregulated genes are marked in light red; downregulated genes are marked in light green. The node size is based on the degree value.

The flow chart of this study is shown in Figure 1. After data standardization and differential expression analysis, the DEGs of each dataset were identified, with 1860 in GSE2503, 1649 in GSE45164, and 1990 in GSE66359. The volcano and heatmaps are shown in Figure 2. As shown in Figure 3(a), a total of 146 common DEGs, including 113 upregulated genes and 33 downregulated genes, were finally identified between cSCC tissues and normal tissues.

### 3.2. Enrichment Analyses of DEGs

**Figure 4:**
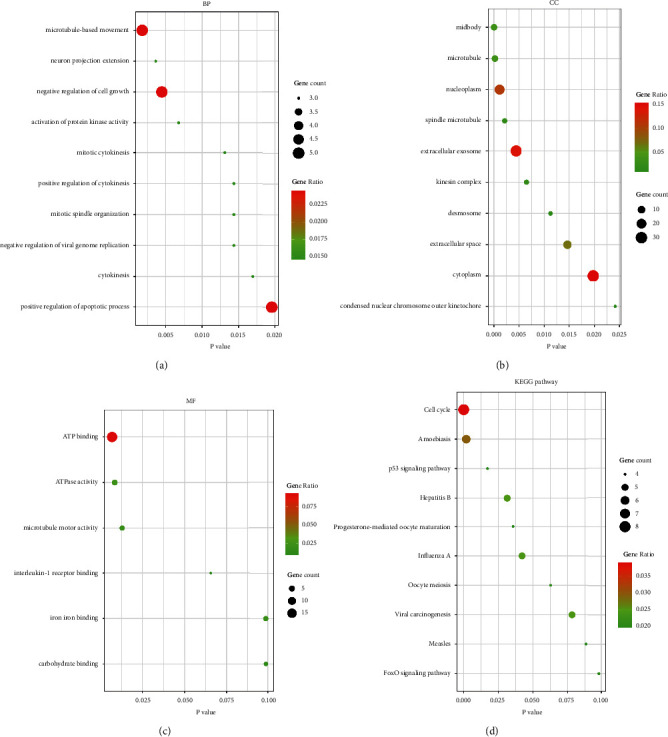
GO and KEGG enrichment analysis of the DEGs. (a) The top 10 enriched GO categories of biological process (BP). (b) The top 10 enriched GO categories of cellular component (CC). (c) The top 6 enriched GO categories of molecular function (MF). (d) A total of 10 signaling pathways in the KEGG enrichment. The abscissa represents the *p* value, and the ordinate represents the terms. The size of the circle represents the number of genes involved, and the color represents the frequency of the genes involved in the term total genes.

The GO analysis results showed that for BP, the DEGs were significantly enriched in microtubule-based movement, negative regulation of cell growth, and positive regulation of apoptotic process (Figure 4(a)). Regarding CC, the DEGs were mainly concentrated in the nucleoplasm, extracellular exosome, and cytoplasm (Figure 4(b)). In terms of the MF, the DEGs mainly focused on ATP binding, ATPase activity, and microtubule motor activity (Figure 4(c)). KEGG pathway analysis showed that the DEGs were mainly concentrated in the cell cycle, p53 signaling pathway, oocyte meiosis, and progesterone-mediated oocyte maturation (Figure 4(d)).

### 3.3. PPI Network Construction and Module Analysis

**Figure 5:**
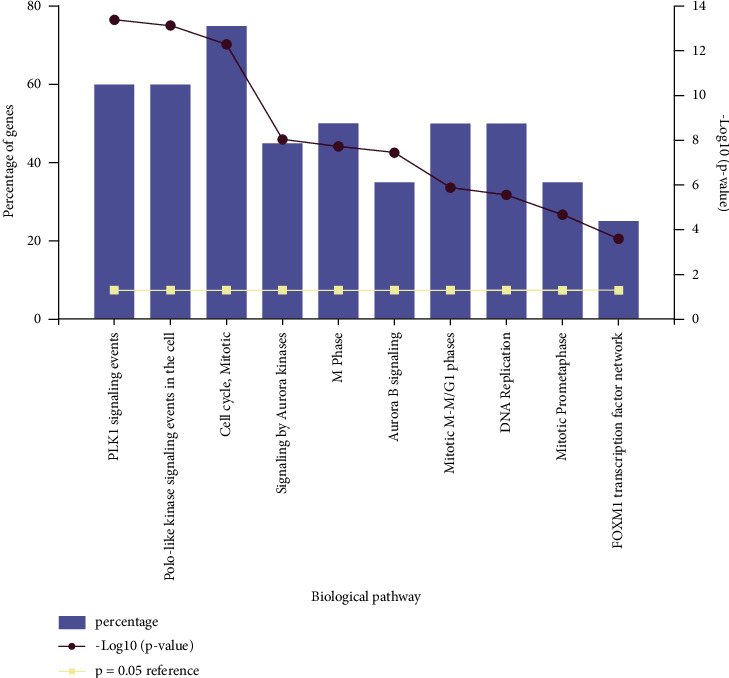
The FunRich software drew a bar chart of five biological pathways based on the *p* value and the percentage of genes, among which biological pathways with *p* value <0.05 are statistically significant.

The PPI network contained 109 nodes and 617 interaction pairs (Figure 3(b)). The most significant module (score = 28.857) was aggregated from the PPI network (Figure 3(c)), including 29 nodes and 404 interaction pairs. Then, when it was put into FunRich for further functional analysis, all genes in this module were upregulated and the enriched biological pathway for the module showed that the DEGs were mainly enriched in *PLK1* signaling events and polo-like kinase signaling events in the cell cycle (Figure 5).

### 3.4. Hub Gene Selection and Analysis

**Figure 6:**
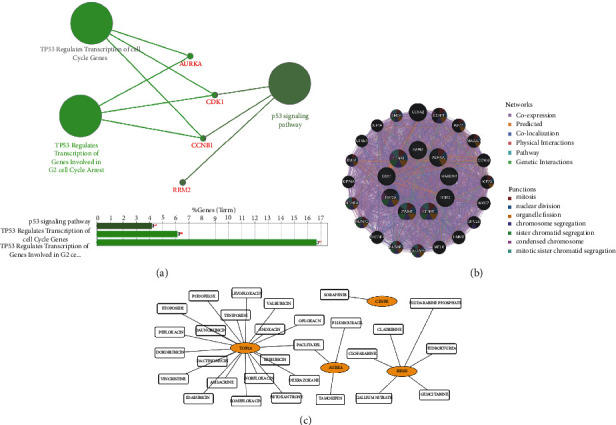
KEGG pathway, coexpression network, and drug‐gene interaction analysis of the hub genes. (a) The most significant pathway and related genes. The results show that these hub genes are mainly involved in the *P53* signaling pathway, *TP53* regulates transcription of cell cycle genes and *TP53* regulates transcription of genes involved in G2 cell cycle arrest. (b) Hub genes and their coexpression genes were analyzed using GeneMANIA. (c) Drug-gene interaction diagram, the yellow circle indicates the differentially expressed gene and the blank square indicates the drug.

A total of nine genes with degrees ≥ 30 were identified as hub genes (details shown in Table 2). ClueGO revealed that the most involved pathways were the *P53* signaling pathway, *TP53* regulates transcription of cell cycle genes and *TP53* regulates transcription of genes involved in G2 cell cycle arrest (Figure 6(a)). The interaction network between hub genes and their coexpressed genes is shown in Figure 6(b). These nine genes showed a complex DEG PPI network with coexpression of 72.69%, prediction of 22.58%, colocalization of 1.86%, physical interactions of 1.73%, a pathway of 1.12%, and genetic interactions of 0.02%. Based on the DGIdb database, we obtained 30 drug‐gene interaction pairs, including four upregulated genes (*AURKA*, *RRM2*, *CENPE,* and *TOP2A*) and 29 drugs (Figure 6(c)).

### 3.5. Validation of Hub Genes in Other Databases and the Human Protein Atlas

**Figure 7:**
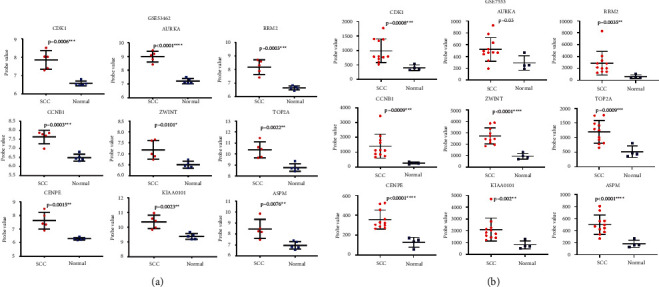
Hub genes expression in the GSE53462 and GSE7553 datasets. SCC stands for cutaneous squamous cell carcinoma tumor tissue and normal represented corresponding normal tissue. ^*∗*^*p* < 0.05; ^*∗∗*^*p* < 0.01; ^*∗∗∗*^*p* < 0.001; ^*∗∗∗∗*^*p* < 0.0001.

**Figure 8:**
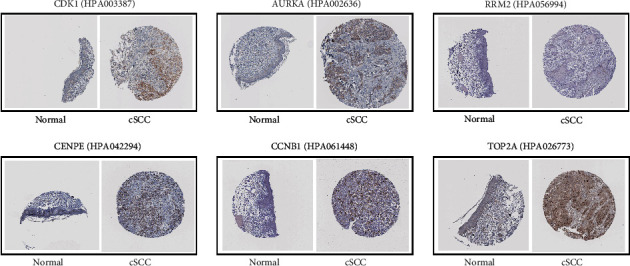
Immunohistochemical expression results of six hub genes from Human Protein Atlas.

Finally, the results of the independence testing analysis suggested that all hub genes were significantly increased in cSCC tumor tissue compared to normal skin tissue (Figure 7). By searching the Human Protein Atlas, we obtained immunohistochemically stained tissue images of six out of the nine hub genes in normal skin tissue and tumor tissue. The results indicated that the six hub genes were significantly differentially expressed between normal and tumor tissues (Figure 8).

### 3.6. TF-miRNA-mRNA Regulatory Network Analysis

**Figure 9:**
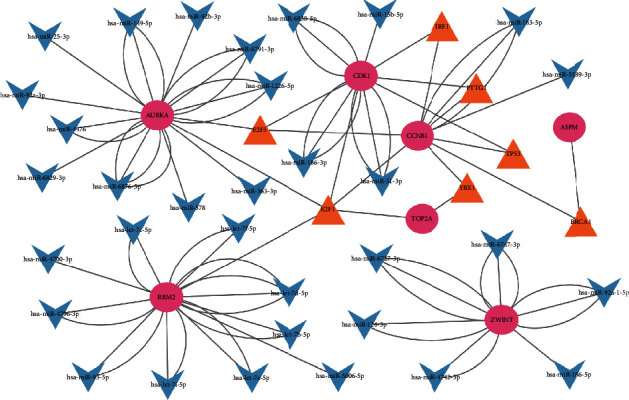
The TF-miRNA-mRNA regulatory network. Red nodes represent hub genes, blue inverted triangles represent miRNAs and yellow triangles represent TFs.

Based on the TRRUST and Mirwalk databases, we found that seven TFs and 33 miRNAs may regulate the expression of these genes. Twenty-nine miRNA-mRNA pairs and 18 TF-mRNA pairs were integrated to construct a TF-miRNA-mRNA regulatory network (Figure 9).

### 3.7. Identification and Validation of Ferroptosis-Related DEGs in cSCC

**Figure 10:**
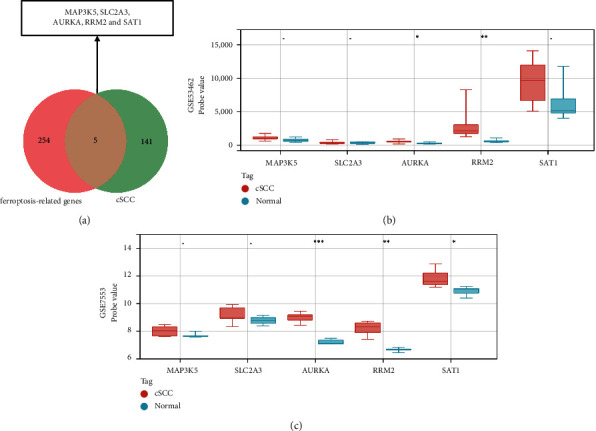
(a) Venn diagram of preliminary ferroptosis-related DEGs in cSCC. ((b)-(c)) The expression levels of preliminary ferroptosis-related DEGs of cSCC in GSE53462 and GSE7553. ^*∗*^*p* < 0.05; ^*∗∗*^*p* < 0.01; ^*∗∗∗*^*p* < 0.001; ^*∗∗∗∗*^*p* < 0.0001.

DEGs in cSCC intersected with 259 ferroptosis-related genes and five genes, were screened. All five genes were upregulated DEGs, including *MAP3K5*, *SLC2A3*, *RRM2*, *AURKA,* and *SAT1*. Subsequently, the expression of these genes was verified in GSE53462 and GSE7553. Especially, *RRM2*, *AURKA,* and *SAT1* were determined to be significant ferroptosis-related genes in cSCC (Figure 10).

## 4. Discussion

cSCC shows the potential for recurrence and metastasis, making it the main cause of death in NMSC [25]. Previous reports have confirmed that mutations in *P53*, *CDKN2A*, *RAS*, *NOTCH1,* and *NOTCH2* are closely related to cSCC [6–9, 26]; however, the underlying molecular mechanisms behind the aggressive progression of cSCC subpopulations remain to be unveiled, which might account for the high mortality rate of cSCC in NMSC [27]. In such a context, both potential and efficient markers for diagnosis and treatment are urgently needed.

In the current study, through analysis of a large sample of cSCC and corresponding normal tissues, 146 DEGs were identified, including 113 upregulated genes and 33 downregulated genes. The upregulated DEGs were mainly enriched in the cell cycle, the *P53* signaling pathway, and oocyte meiosis. According to previous studies, disorders of the cell cycle process play an important role in the development of tumors [28] and the *P53* signaling pathway is closely related to the progression of cSCC [29]. In addition, the biological pathway for the most significant module showed that the DEGs were mainly enriched in *PLK1* signaling events and polo-like kinase signaling events in the cell cycle. Previous studies have confirmed that by inhibiting cSCC keratinocyte *PLK1* signaling in vitro, the cancer cells die first, emphasizing the indispensability of the *PLK1* signaling pathway in the development of cSCC [30]. In this regard, our results were consistent with all of these theories.

A total of nine genes were identified as hub genes with degrees ≥ 30, namely, *CDK1*, *AURKA*, *RRM2*, *CENPE*, *CCNB1*, *KIAA0101*, *ZWINT*, *TOP2A*, and *ASPM*. These genes were verified in the GSE53462 dataset. Among these genes, there are four druggable genes, including *AURKA*, *RRM2*, *CENPE,* and *TOP2A*. *AURKA* is one of three members of the highly conserved mitogen kinase family and it plays an essential role in regulating cell division, which is necessary for timely access to mitosis, centrosome maturation, and the assembly of bipolar spindles [31]. Previous studies have found that the expression levels of *AURKA* in squamous cell carcinoma and adenocarcinoma are significantly different [32]. In addition, Torchia et al. established a mouse model of *AURKA* overexpression, suggesting that *AURKA* has a clear role in the malignant progression of cSCC [33]. The overexpression of *RRM2* significantly enhances the invasiveness of the cells and plays a key role in determining the degree of tumor malignancy [34, 35]. However, the role of *RRM2* in the development of cSCC is unclear. The protein encoded by *CENPE* is a forward-directed kinesin belonging to the kinesin-7 subfamily, which has a critical role in mitosis [36]. Increasing evidence has shown that *CENPE* may be a useful drug target for several tumors without targeted therapy [37]. Recent studies have confirmed that *CENPE* is highly expressed in lung adenocarcinoma tissues and promotes lung adenocarcinoma cell proliferation [38]. Meanwhile, *TOP2A* encodes DNA topoisomerase and is involved in important cellular functions such as DNA replication, transcription, recombination, and mitosis. It is a sign of cell proliferation in normal and tumor tissues. High expression of *TOP2A* occurs most often in breast cancer, where it is strongly correlated with the patients' disease-free survival and total survival, and thus it is regarded as a valuable prognostic biomarker for breast cancer [39–41]. In addition, high expression of *TOP2A* was related to the cell cycle, and targeting *TOP2A* is also considered to be an important method for treating human cancer [42]. In summary, these genes play significant roles in cSCC.

Studies have shown that *CDK1* is overexpressed in breast cancer and liver cancer, causing tumor cell proliferation and development [43, 44]. In addition, *CDK1* is a marker of the clinical prognosis of colon cancer [45]. *CCNB1* is a member of the cyclin family. *CCNB1* and *CDC2* combine to form an M-phase promoting factor (MPF), which promotes cells from the G2 to the M phase [46]. Overexpression of *CCNB1* damages the cell's G2/M detection point and causes an increase in MPF. DNA damage cannot be detected, and mitosis still occurs, causing the proteasome to break down and recognize the MPFs only during the middle stage of division, resulting in the continuous proliferation and development of tumor cells [47]. Therefore, *CCNB1* dysregulation allows cancer cells to proliferate and differentiate, and the new cancer cells promote the expression of *CCNB1* to increase further [48]. Moreover, previous studies have reported that *KIAA0101* overexpression in mammalian cells can prevent UV-induced apoptosis, suggesting it has a protective effect in regulating DNA repair, cell proliferation, apoptosis, and cell cycle progression [49]. *KIAA0101* is closely related to the invasion and metastasis of cancer cells [50]. However, no one has studied its role in cSCC. *ZWINT* is another centromere complex component required for mitotic spindle checkpoints, and it is involved in centromere function and cell growth [51]. Recently, *ZWINT* overexpression has been reported in ovarian cancer and hepatocellular carcinoma, and it is intimately linked to tumor progression and a poor prognosis [52, 53]. In addition, *ASPM*, as a cell cycle progression gene, is a key factor in mitotic spindle regulation [54]. Previous studies have shown that *ASPM* is highly expressed in ovarian, pancreatic, and prostate cancers and it is significantly associated with a poor prognosis [55–57]. According to recent findings, knockout of *TPX2* in prostate cancer can induce cell cycle quiescence and apoptosis, reduce the ability of cells to invade, and inhibit cell proliferation [58].

Previous studies have mainly focused on the common DEGs between AK and cSCC tissues to confirm that AK is a precursor lesion of cSCC [59]. In addition, a group of epithelial-mesenchymal transition (EMT) and autophagy-related genes involved in cSCC was discovered, and the results showed that inhibition of autophagy and activation of EMT played important roles in the development of cSCC [59]. In this study, we identified and validated ferroptosis-related DEGs in cSCC to reveal the potential mechanism, which may provide a new direction for exploration. Ferroptosis is a novel iron-dependent type of programmed cell death different from apoptosis, necrosis, and autophagy [60]. Previous studies have reported that ferroptosis in squamous cell carcinoma is closely related to cancer progression [61–64]. However, the mechanism and role of ferroptosis in cSCC have rarely been reported in the literature. Among the ferroptosis-related hub genes, *RRM2* was downregulated in cells treated with the ferroptosis inducer erastin, suggesting that ferroptosis may be inhibited in a GSH-dependent manner [65]. Similarly, inhibition of *AURKA* or reconstitution of miR-4715-3p inhibited *GPX4* and induced cell death, suggesting a link between *AURKA* and ferroptosis [66]. Additionally, *P53*-mediated activation of *SAT1* contributes to ferroptotic cell death in the presence of ROS stress. Knockdown of *SAT1* partially rescued ROS-induced ferroptosis [67]. These studies suggest that the ferroptosis-related genes we identified may play an important role in the development of cSCC. However, a more in-depth study of the mechanism of ferroptosis in cSCC is urgently needed.

We would like to acknowledge the limitations of this research. First, this was a retrospective study. All of the data in this study come from publicly available databases. Second, further in vivo and in vitro experiments are required to confirm these results. Third, we must further study the underlying mechanism of signaling pathways in cSCC.

## 5. Conclusion

In summary, the purpose of this study was to explore the underlying molecular mechanism of cSCC. A total of nine hub genes were identified, including *CDK1*, *AURKA*, *RRM2*, *CENPE*, *CCNB1*, *KIAA0101*, *ZWINT*, *TOP2A,* and *ASPM*. Among these genes, there are four druggable genes, including *AURKA*, *RRM2*, *CENPE,* and *TOP2A*. In addition, *RRM2*, *AURKA,* and *SAT1* were identified as significant ferroptosis-related genes in cSCC. The above findings provide potential research directions and drug targets for cSCC research. In conclusion, *AURKA* and *RRM2* should be the focus of future research. Further mechanistic and drug development research on cSCC is necessary.

## Figures and Tables

**Table 1:** Details of the GEO datasets.

Dataset	Platform	No. of samples (cSCC vs. HC)
GSE2503	GPL96[HG-U133A] Affymetrix Human Genome U133A Array	5, 6
GSE45164	GPL571 [HG‐U133A_2] Affymetrix Human Genome U133A 2.0 Array	10, 3
GSE66359	GPL570 [HG‐U133_Plus_2] Affymetrix Human Genome U133 Plus 2.0 Array	8, 5
GSE53462	GPL10558 Illumina Human HT-12 V4.0 Expression BeadChip	5, 5
GSE7553	GPL570 [HG-U133_Plus_2] Affymetrix Human Genome U133 Plus 2.0 Array	11, 4

**Table 2:** Functional roles of nine hub genes with degree ≥ 30.

Gene symbol	Degree	Full name	Function
*CDK1*	35	Cyclin-dependent kinase 1	*CDK1* can regulate the cell cycle progression, apoptosis, and carcinogenesis of tumor cells
*AURKA*	32	Aurora kinase A	*AURKA* is one of three members of the highly conserved mitogen kinase family and plays an important role in regulating cell division.
*RRM2*	32	Ribonucleotide reductase regulatory subunit M2	*RRM2* can enhance the invasiveness of the cells and played a key role in determining the degree of tumor malignancy.
*CENPE*	31	Centromere protein E	*CENPE* may be a useful drug target for several tumors without targeted therapy.
*CCNB1*	31	Cyclin B1	*CCNB1* and squamous cells play a complementary role, allowing cancer cells to further proliferate and differentiate.
*KIAA0101*	30	*KIAA0101*	*KIAA0101* is closely related to the invasion and metastasis of cancer cells.
*ZWINT*	30	ZW10 interacting kinetochore protein	*ZWINT* is a centromere complex component required for mitotic spindle checkpoints and is involved in centromere function and cell growth.
*TOP2A*	30	Topoisomerase (DNA) II alpha	This gene encodes a DNA topoisomerase, an enzyme that controls and alters the topologic states of DNA during transcription. *TOP2A* acts as a target for several anticancer agents and mutations of this gene have been associated with drug resistance
*ASPM*	30	Abnormal spindle microtubule assembly	*ASPM*, as a cell cycle progression gene, plays a critical role in mitotic spindle regulation.

## Data Availability

In this study, mRNA microarray datasets were downloaded from the Gene Expression Omnibus (https://www.ncbi.nlm.nih.gov/geo).

## Abbreviations

cSCC: Cutaneous squamous cell carcinoma
IHC: Immunohistochemistry
AK: Actinic keratosis
GEO: Gene expression omnibus
DEGs: Differentially expressed genes
PPI: Protein-protein interaction network
BCC: Basal cell carcinoma
NMSC: Nonmelanoma skin cancers
FC: Fold change
GO: Gene ontology
BP: Biological processes
MF: Molecular functions
CC: Cell composition
MPF: M-phase promoting factor
EMT: Epithelial-mesenchymal transition.
